# Supplementary material for: Possibilities of Using Medicinal Plant Extracts and Salt-Containing Raw Materials from the Aral Region for Cosmetic Purposes
Source: Molecules. 2022 Aug 11;27(16):5122. doi: 10.3390/molecules27165122 (PMC9416250; doi:10.3390/molecules27165122)
Supplement: Supplementary file 1 [file molecules-27-05122-s001.zip › molecules-1686686-supplementary.pdf]

Article

Possibilities of Using Medicinal Plants Extracts and Salt-Containing Raw Materials from the Aral Region for Cosmetic Purposes

Izabela Nowak [1,*], Akmaral Issayeva [2,*], Marta Dąbrowska [1], Agata Wawrzyńczak [1], Henryk Jeleń [3], Bogusława Łęska [1], Azhar Abubakirova [4] and Assel Tleukeyeva [4]

1 Faculty of Chemistry, Adam Mickiewicz University, Uniwersytetu Poznańskiego 8, 61-614 Poznań, Poland
2 Ecology and Biology Research Institute, Shymkent University, Shymkent 160000, Kazakhstan
3 Faculty of Food Science and Nutrition, Poznań University of Life Sciences, Wojska Polskiego 31, 60-624 Poznań, Poland
4 M. Auezov South Kazakhstan University, Shymkent 160000, Kazakhstan
* Correspondence: nowakiza@amu.edu.pl (I.N.); akmaral.issayeva@bk.ru (A.I.)

Suplementary Materials

Sample preparation

***Scrub* 1.** Universal scrub for normal to oily skin types. The scrub is based on: the muddy bottom mud of the Aral Sea - 80%, the scarifier (fine grinding of walnut shells) -4%, herbal extracts -9%, and natural aromatic additives -7%. The composition of medicinal herbs contains the following composition,% by weight:

- grinding of ordinary harmonica –0.5;
- grinding of tansy –0.5;
- grinding of thyme ordinary –1.0;
- grinding of sage meadow –2.0;
- grinding of yarrow -1.0;
- grinding of licorice -4.0;
- grinding the mountaineer -4.0.

Natural aromatic additives contain the following composition,% by weight:

-extract of essential oils of garden roses -2.0;
- extract of essential oils of thyme ordinary -5.0.

***Scrub* 2.** Scrub for sensitive skin. The basis of the scrub is the muddy bottom mud of the Aral Sea -75%. As a scarifier, grinding of phytomass of medicinal plants after extraction of -15% is used. Herbal extract 8%, natural aromatic additives -2%. The composition of medicinal herbs contains the following composition,% by weight:

- grinding of licorice -3.0;
- grinding of white mulberry -1.0;
- grinding of silver sucker - 1.0;
- grinding of the mountaineer -3.0;
- grinding of mint field t - 1.0;

- grinding of lemon balm 1.0;
- grinding the petals of a garden red rose -2.

Natural aromatic additives contain the following composition,% by weight:

silver sucker essential oil extract -1.0;

extract of essential oils of mint field - 1.0.

Scrub 3. Healing scrub for problem skin without the content of hard scarifiers. The basis of the scrub is healing mud mud of the Aral Sea -80. As a scarifier, grinding phytomass of a special composition of medicinal plants after extraction of -14% is used. Herbal extract 6%, natural aromatic additives -2%. The composition of medicinal herbs contains the following composition,% by weight:

- grinding of thyme ordinary -2.0;
- grinding of sage meadow - 1.0;
- grinding of lemon balm medicinal - 5.0;
- grinding of white mulberry -2.0;
- grinding of yarrow -2.0;
- grinding of psoralea skeleton - 4.0;
- grinding of the camel thorns -5.0.

Natural aromatic additives contain the following composition,% by weight:

- sage essential oil extract -1.0;
- extract of thyme essential oils -1.0.

Universal mask 4. The basis of the mask is healing mud of the Aral Sea -70%. Grinding and extract of herbs -20%, natural aromatic additives - 10%. The composition of medicinal herbs contains the following composition,% by weight:

- grinding sage meadow -3.0;
- grinding of thyme ordinary-6.0;
- grinding of yarrow -3.0;
- grinding of ordinary harmonica -2.0;
- grinding of camel thorns -2.0;
- grinding of lemon balm medicinal -2.0;
- grinding of wormwood citrate -2.0;
- grinding of a red garden rose -3.0.

Natural aromatic additives contain the following composition,% by weight:

- extract of essential oils of sage meadow -3.0;
- extract of essential oils of thyme ordinary -2.0.

Mask 5. Mask for sensitive skin for dry and problematic skin types. The basis of the mask is healing mud of the Aral Sea -83%. Grinding and extract of herbs -12%, natural aromatic additives - 5%. The composition of medicinal herbs contains the following composition,% by weight:

- grinding of yarrow -1.0;
- grinding of mint field mint -6.0;
- grinding of lemon balm -1.0;

- grinding of silver sucker -2.0;
- grinding of petals of a white garden rose -1.0;
- grinding of white mulberry-6.0.

Natural aromatic additives contain the following composition,% by weight:

- extract of essential oils of common lilac -3.0;

black elderberry essential oil extract -2.0.

Mask 6. The mask is medical. The basis of the mask is healing mud of the Aral Sea - 72%. Grinding and extract of medicinal herbs 25-%, natural aromatic additives - 3%. The composition of medicinal herbs contains the following composition,% by weight:

- grinding of psoralean skeleton -20.0;
- grinding of white mulberry - 3.0;
- grinding petals of a white garden rose - 2.0.

Natural aromatic additives contain the following composition,% by weight:

-extract of essential oils of lemon balm -3.0.

Scrub 7. Nutritious scrub. The basis of the scrub is healing mud mud of the Aral Sea - 90%. Grinding and extract of herbs -7%, natural aromatic additives - 3%. The composition of medicinal herbs contains the following composition,% by weight:

- grinding of sage meadow -3.0;
- grinding of thyme ordinary -3.0;
- grinding of yarrow -3.0;
- grinding of ordinary harmonica -2.0;
- grinding of camel thorns -2.0;
- grinding of lemon balm medicinal -2.0;
- grinding of wormwood citrate -2.0;
- grinding of a red garden rose -3.0.

Natural aromatic additives contain the following composition,% by weight:

- extract of essential oils of sage meadow -3.0;
- thyme essential oil extract -2.0

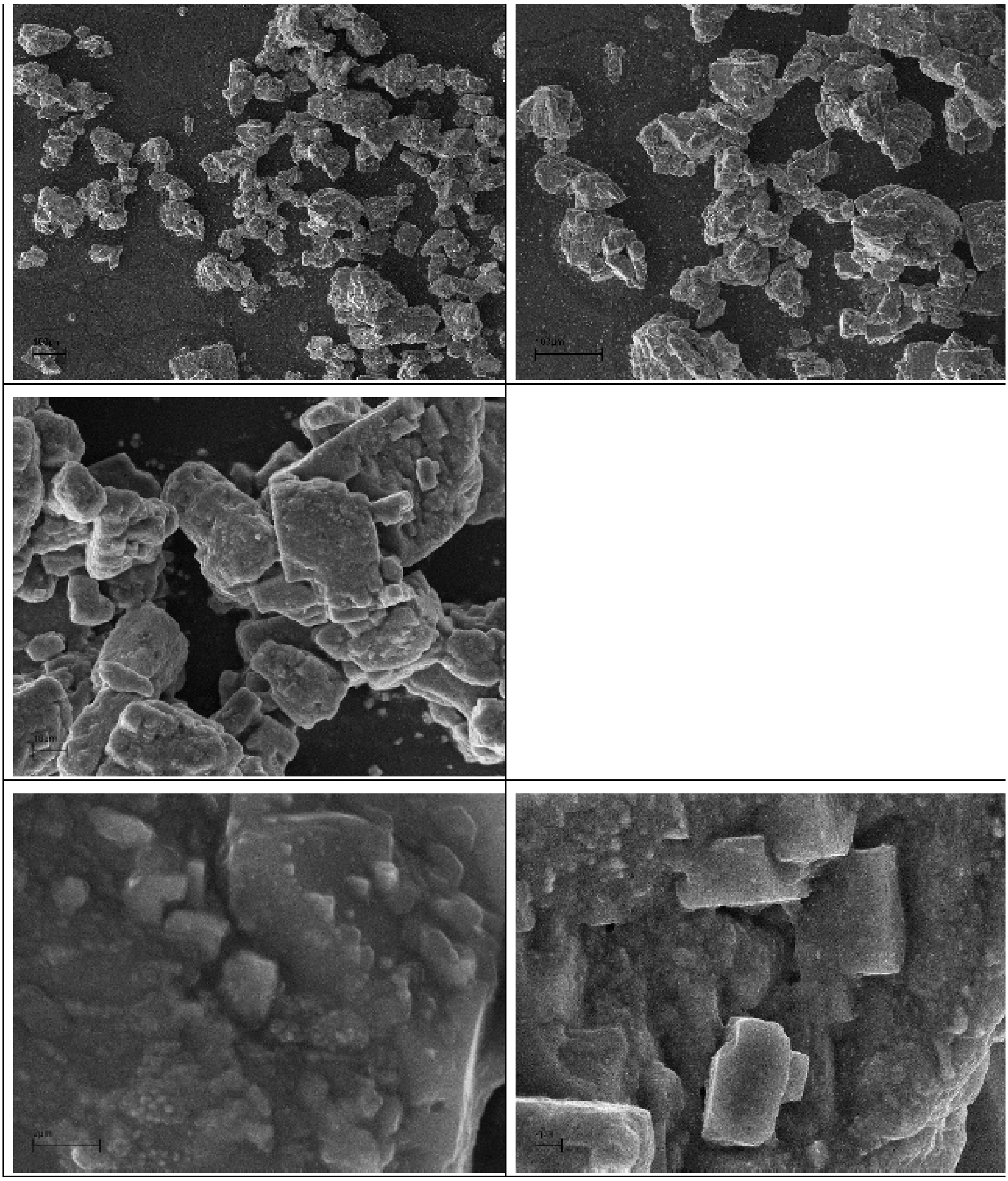

Figure S1. A. SEM images of sample S1.

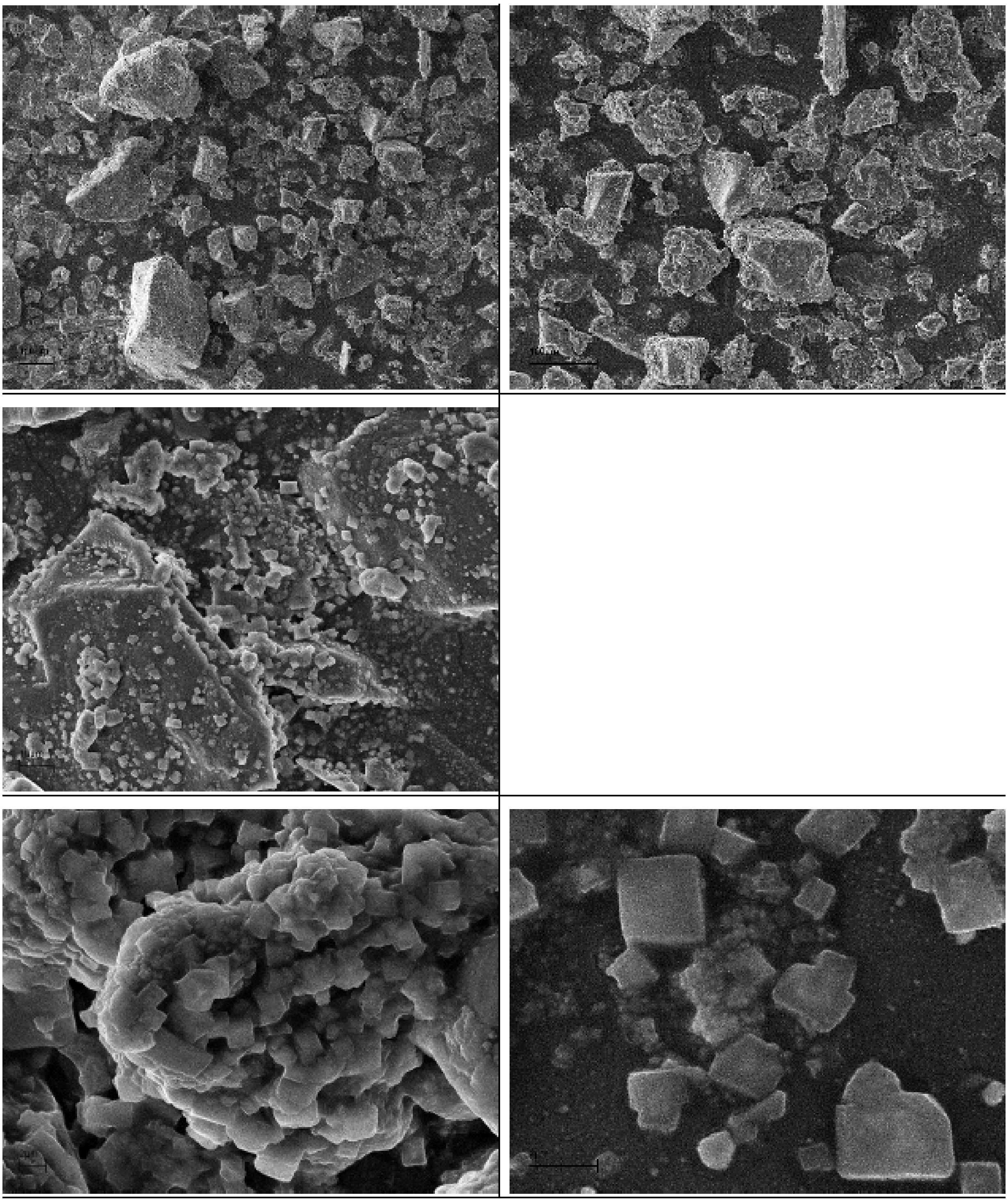

Figure S1. B. SEM images of sample S2.

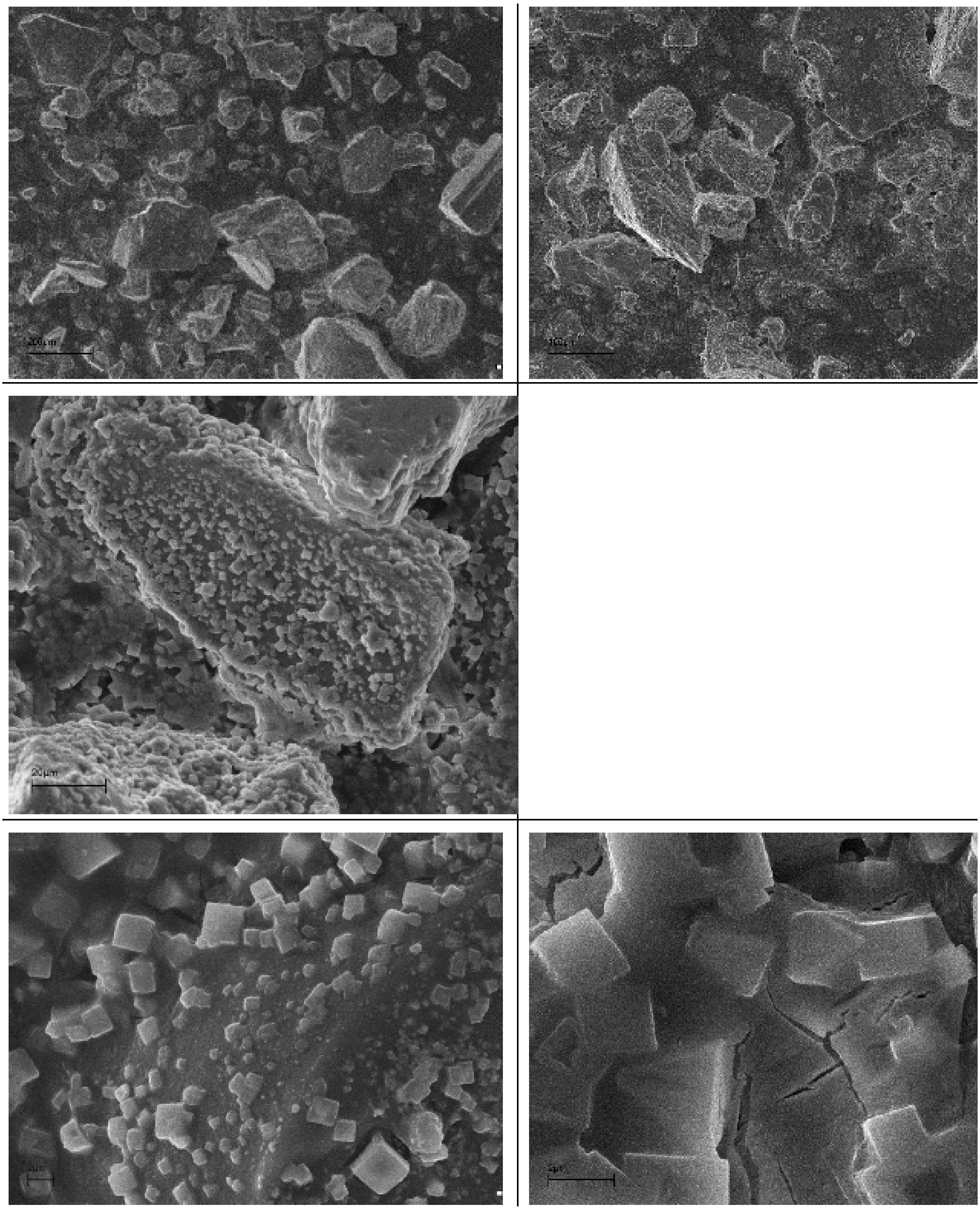

Figure S1. C. SEM images of sample S3.

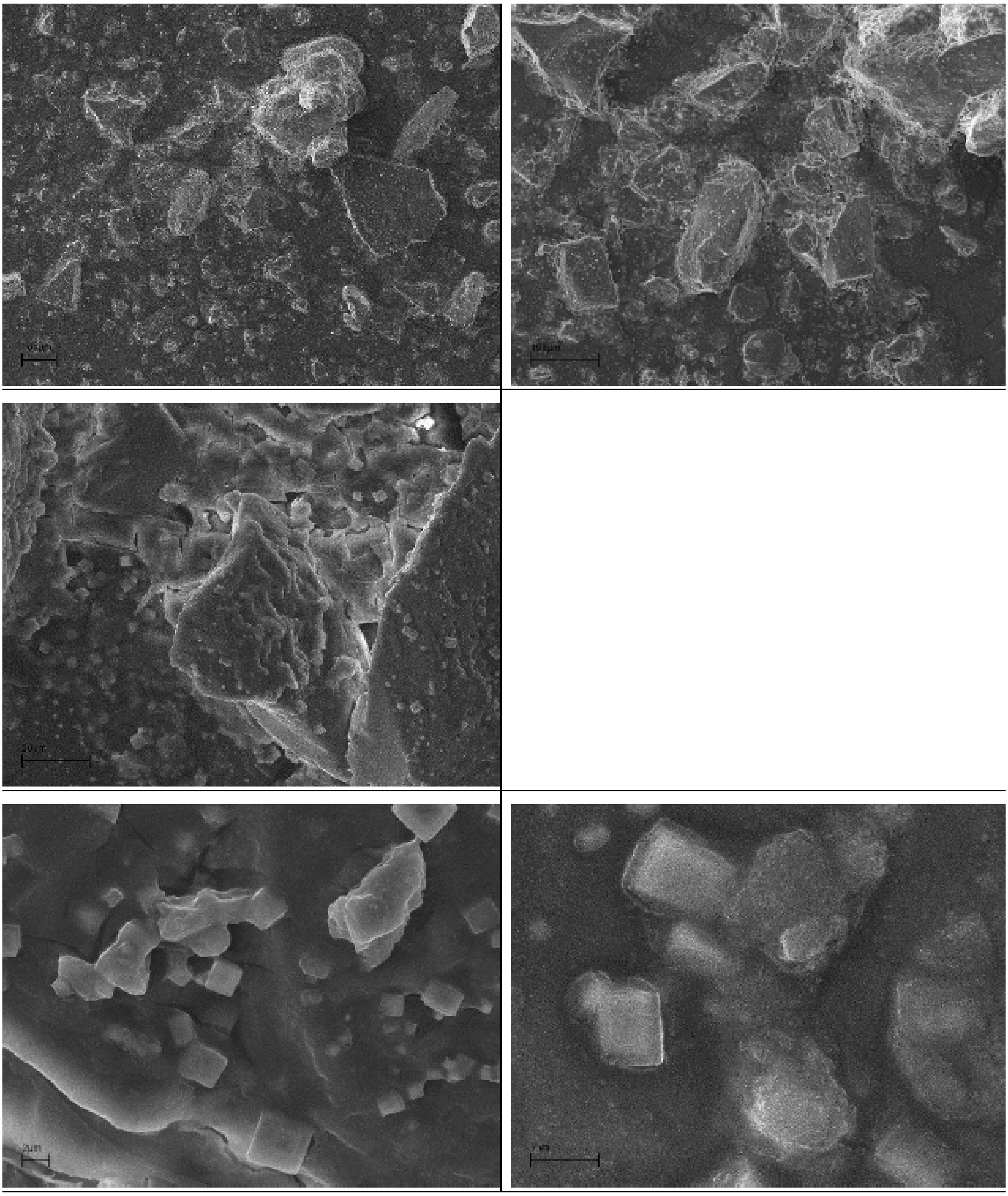

Figure S1. D. SEM images of sample S1.

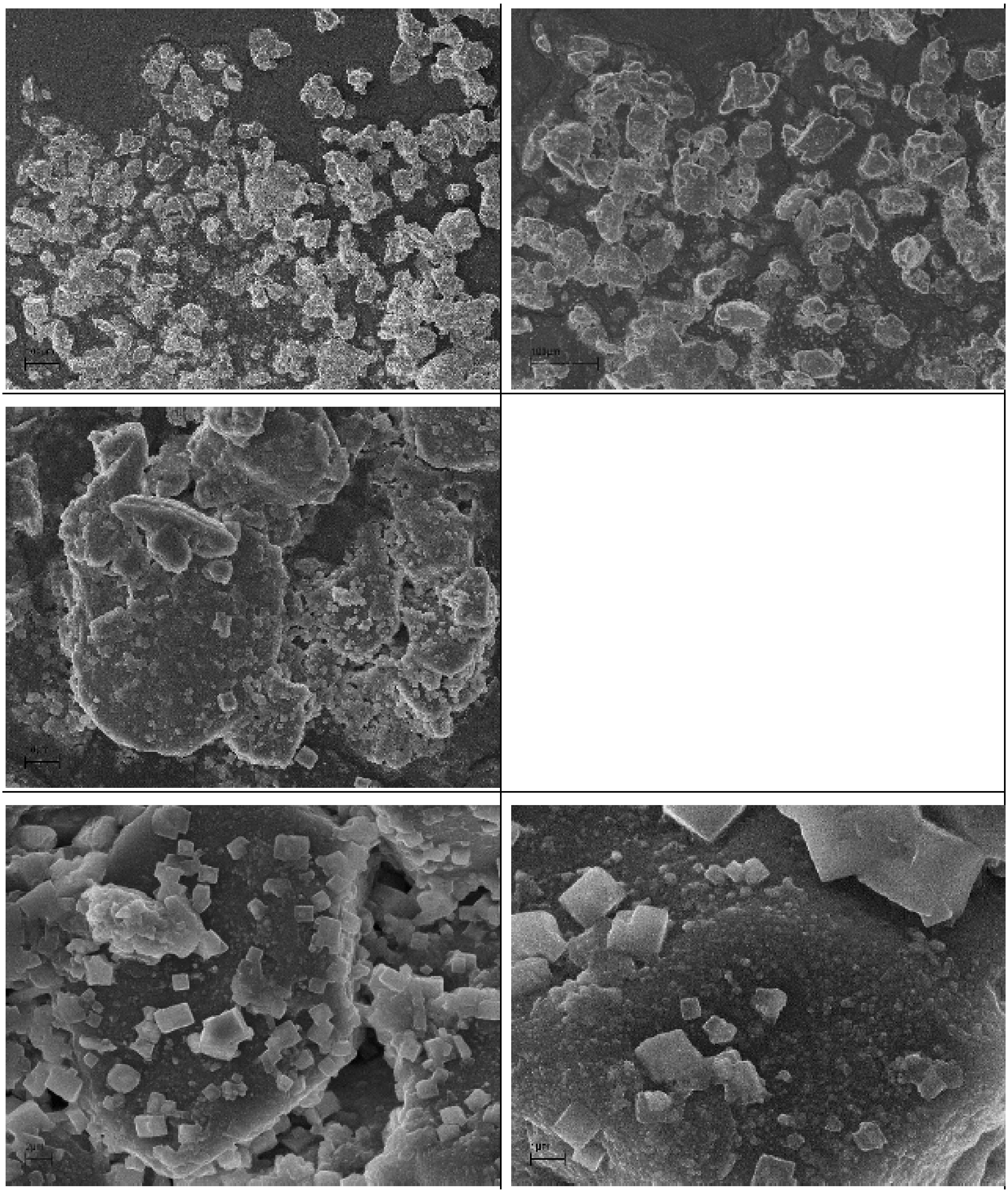

Figure S1. E. SEM eimages of sample S1.

Figure S1. SEM images

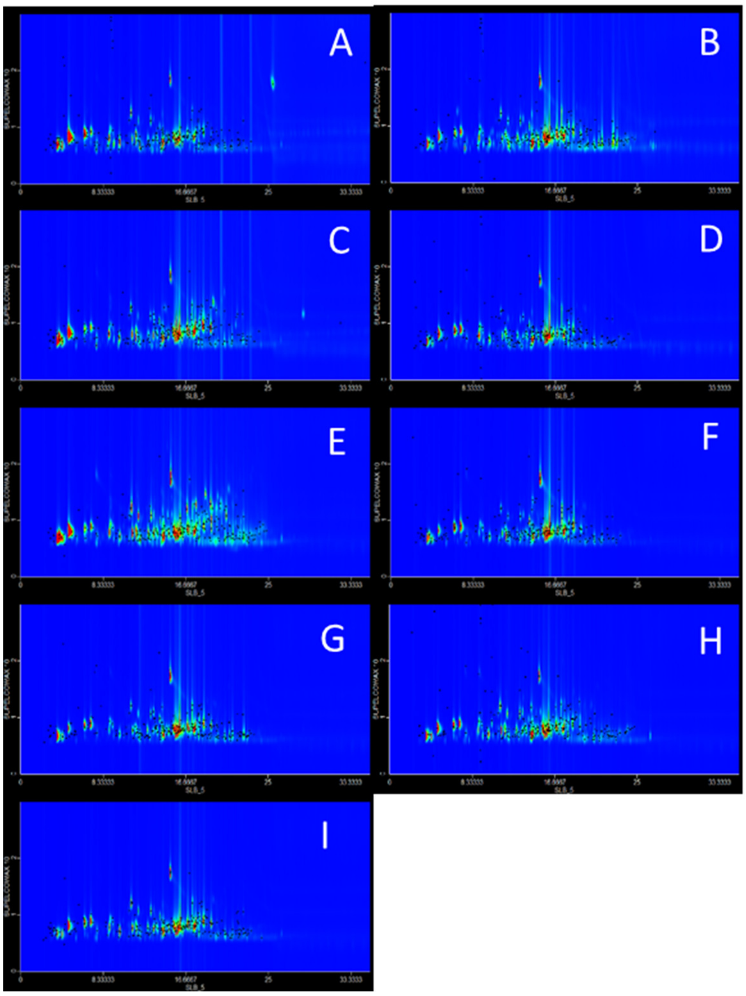

Figure S2. TIC chromatograms of volatile compounds isolated from plants discussed in the manuscript.

Scrub 1

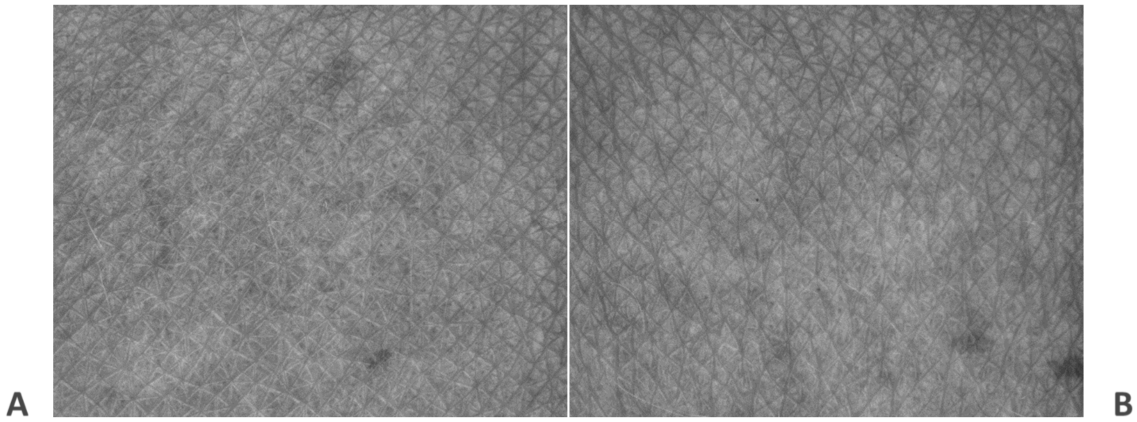

SEr	SEsm	SEsc	SEw	SEr	SEsm	SEsc	SEw
2.32	84.68	0.65	28.569	1.95	105.24	0.56	39.049

Scrub 2

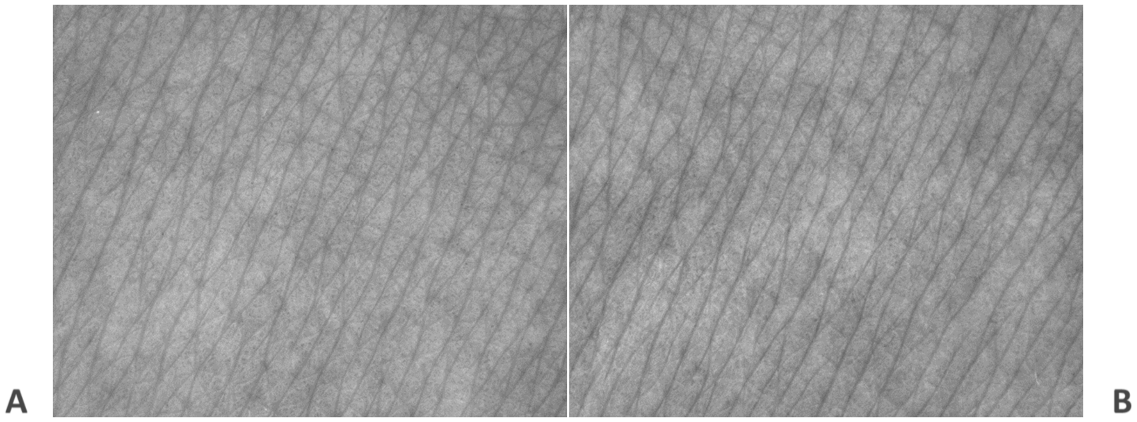

SEr	SEsm	SEsc	SEw	SEr	SEsm	SEsc	SEw
2.11	98.29	0.51	37.803	2.19	112.85	0.62	43.810

Mask 5

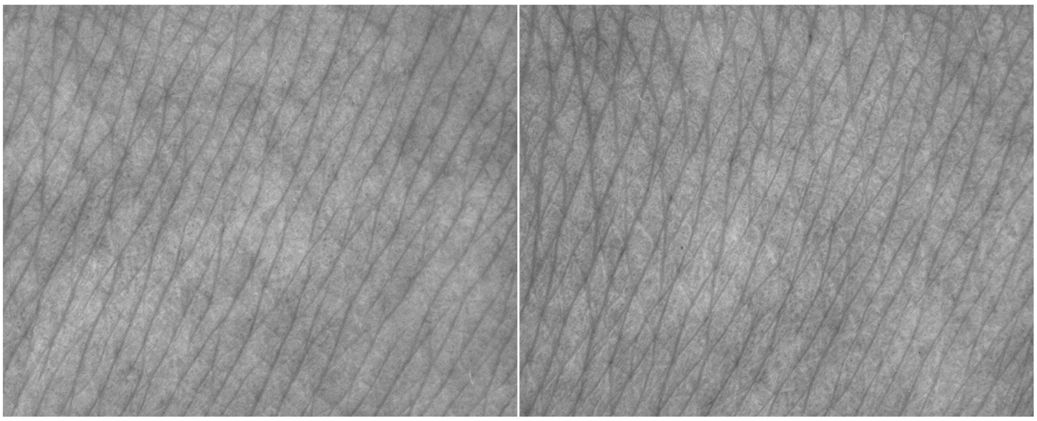

A B

SEr	SEsm	SEsc	SEw	SEr	SEsm	SEsc	SEw
2.19	112.85	0.62	43.810	2.72	114.80	0.50	24.944

Mask 6

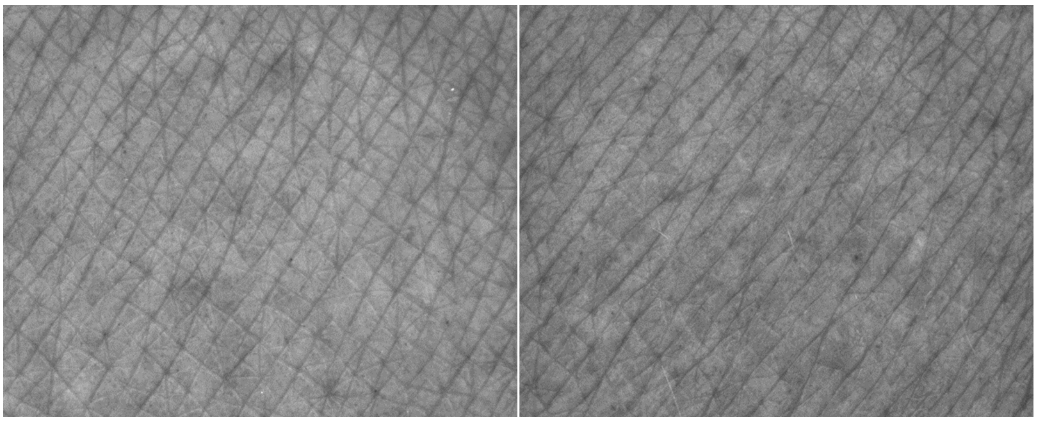

A B

SEr	SEsm	SEsc	SEw	SEr	SEsm	SEsc	SEw
3.32	111.35	0.46	44.089	2.39	104.97	0.59	42.139

Scrub 7

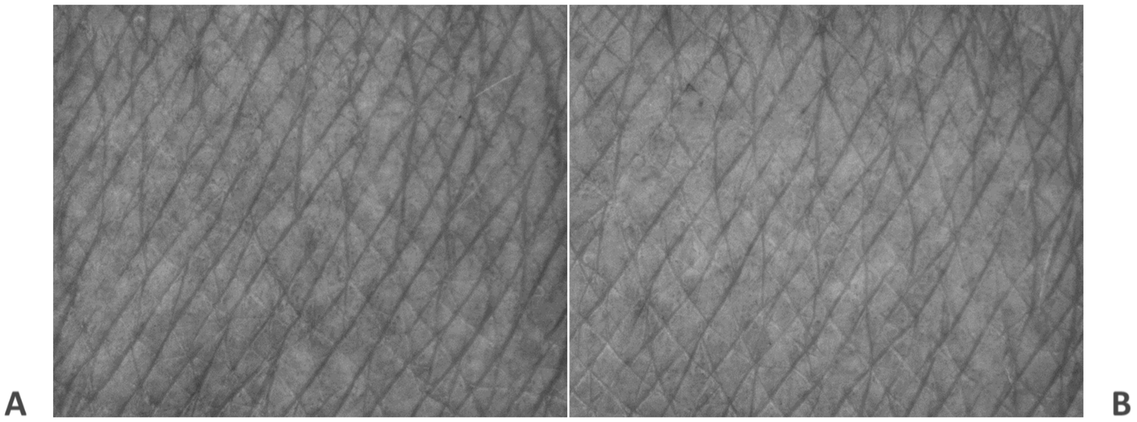

SEr	SEsm	SEsc	SEw	SEr	SEsm	SEsc	SEw
2.14	137.10	0.38	64.426	2.99	131.73	0.54	62.549

Figure S3. Skin topography images obtained before (A) and after application (B) of tested cosmetic products 1, 2, 5-7 with the corresponding parameters of skin topography
